# Association between neutrophil-to-lymphocyte ratio and differentiated thyroid cancer: a meta-analysis

**DOI:** 10.1038/srep38551

**Published:** 2016-12-12

**Authors:** Ji-Feng Liu, Luo Ba, Hong Lv, Dan Lv, Jin-Tao Du, Xiao-Mei Jing, Ning-Jing Yang, Shao-Xin Wang, Chao Li, Xiao-Xia Li

**Affiliations:** 1 Department of Head and Neck Surgery, Sichuan Cancer Hospital, Chengdu, China; 2 Department of Otorhinolaryngology of People’s Hospital of Tibet Autonomous Region, Lhasa, China; 3 Department of Otorhinolaryngology – Head and Neck Surgery, West China Hospital of Sichuan University, Chengdu, China; 4 Department of oncology, Sichuan Cancer Hospital, Chengdu, China; 5 Department of Image, Sichuan Cancer Hospital, Chengdu, China

## Abstract

The association between neutrophil-to-lymphocyte ratio (NLR) and differentiated thyroid cancer (DTC) is undecided. To rectify this question, we conducted a systematic meta-analysis based on 7 prospective cohort studies published between 2013 and 2015, comprising 7349 patients. Six of these cohorts included pretreatment (baseline) NLR data for patients with thyroid nodules. The meta-analysis of these 6 cohorts showed that the NLR of patients with DTC (4617 cases) was statistically similar to patients with benign nodules only (1666 cases), with a mean difference (MD) of 0.19 (95% CI: −0.09 to 0.46; I^2^ = 93%; *P* < 0.001). No significant difference in NLR was found between patients with DTC and patients with benign nodules. Two studies addressed an association between NLR and papillary thyroid carcinoma in patients stratified by age <45 and ≥45 years (496 and 891 cases, respectively); the pooled MD was 0.09 (95% CI: −0.37 to 0.55; I^2^ = 92.2%, *P* < 0.001). An elevated NLR seems not a reliable indicator of progressing DTC in patients with goiters, and there was no difference in NLR between patients aged <45 years and those aged ≥45 years. Well-designed and large-scale investigations are warranted to understand the value of NLR in the prognosis of DTC.

Thyroid nodules are very common, found in more than 50% of patients when using ultrasonography^1^. While the prevalence of thyroid nodules in the population is increasing, only 5% to 10% harbor malignant disease^2^. Still, there is a pressing need to correctly identify the nature of thyroid nodules^3^, because thyroid cancer is the most common endocrine malignancy^4^ and often presents in thyroid nodules. Differentiated thyroid cancers (DTCs) account for approximately 95% of all thyroid cancer and generally have a favorable prognosis^5^. Thyroid cancer is closely associated with inflammation^6^, and several studies suggest that there is a higher incidence of DTC in patients with thyroiditis^7^,^8^.

In a variety of cancers, hematological components of the systemic inflammatory response have been shown to have prognostic value, especially the neutrophil-to-lymphocyte ratio (NLR)^9^. NLR is inexpensive to determine and can be routinely measured in day-to-day oncological practice^9^, and it may be useful for identifying high-risk patients^10^. Consequently, many researchers have explored the association between the NLR and thyroid cancer.

According our literature search, Seretis *et al*.^11^ was the first to report that the preoperative NLR was significantly elevated in patients with papillary thyroid microcarcinomas or thyroid cancer. They proposed that the NLR was an easily accessible biomarker for detecting incidental papillary thyroid microcarcinoma. The studies of Kim *et al*.^12^ and Kocer *et al*.^13^ were in accord with this, but Liu *et al*.’s^14^ only for patients 45 years and older. However, Liu *et al*.^14^ and Kim *et al*.^15^ found that there was no difference in the NLRs between patients with benign or malignant thyroid nodules in the overall population.

Thus, the association between NLR and DTC remains controversial. The conflicting data among the above studies may be due to limited sample sizes, the demographic differences in populations, or both. To explore the potential association between NLR and DTC, we conducted a rigorous systematic meta-analysis of relevant prospective cohort studies.

## Results

### Study selection and characteristics

**Figure 1:**
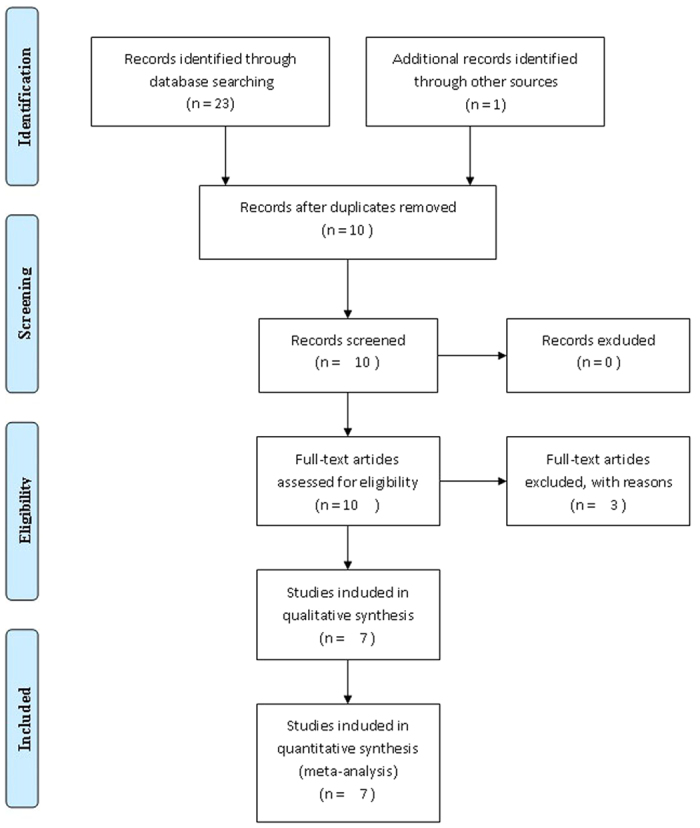
Flow chart of the included studies.

The initial search retrieved 23 studies (Fig. 1). After screening the titles or abstracts, 14 studies were excluded for being duplicate reports or irrelevant. Three more reports were discarded for the following reasons: one did not provide standard deviations of the NLR values of patients with benign nodules or thyroid cancer; 2 failed to provide NLR data for both benign nodules and thyroid cancer. One study was added from a reference list^12^.

Therefore, 7 studies comprising 7349 patients, published between 2013 and 2015, were included in the present meta-analysis^4^,^12^,^13^,^14^,^15^,^16^,^17^ (Table 1). Three studies were conducted in Korea^12^,^15^,^16^,^17^, three studies were performed in China, and one in Turkey^13^. NOS^18^ scores of the studies ranged from 7 to 8, with a mean value of 7.71.

### NLR in patients with DTC or benign nodules

**Figure 2:**
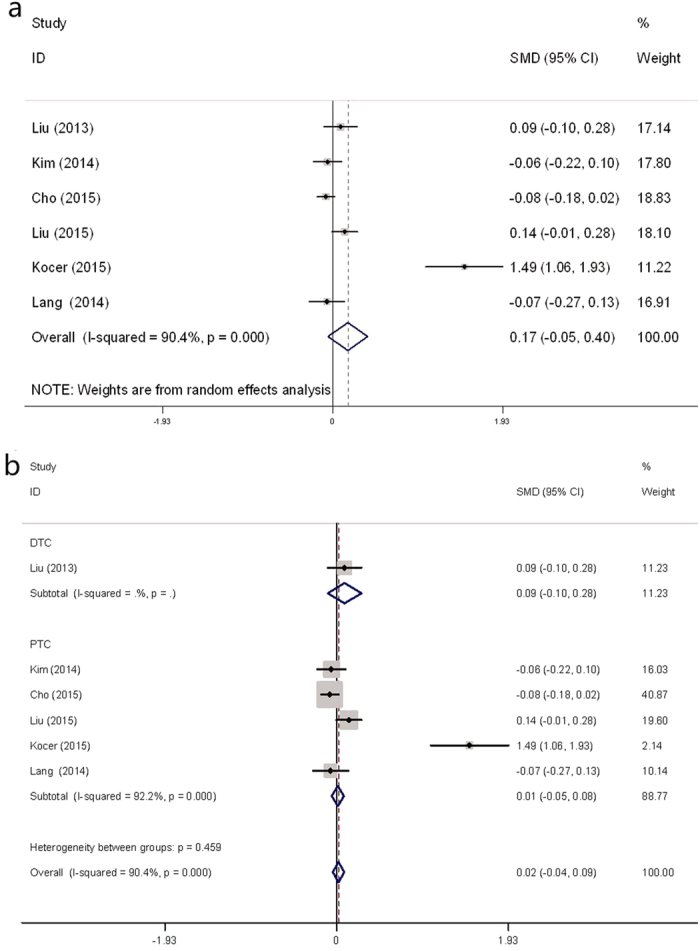
Forest plots of preoperative NLR in DTC patients compared with those with benign nodules. (**a**) NLR difference between DTC and benign nodules in the overall population. (**b**) NLR difference between PTC and benign nodules.

Six studies contained pretreatment (baseline) NLR data for patients with thyroid nodules^4^,^12^,^13^,^14^,^16^,^17^. A meta-analysis of these 6 cohorts showed that the NLR values of patients with DTC and those with benign nodules were statistically similar (mean difference [MD] = 0.19; 95% confidence interval [CI]: −0.09 to 0.46; Fig. 2a), although there was heterogeneity among the studies (Cochran’s Q test and Higgins I-squared statistic [I^2^ ] = 93%, *P* < 0.001).

**Figure 3:**
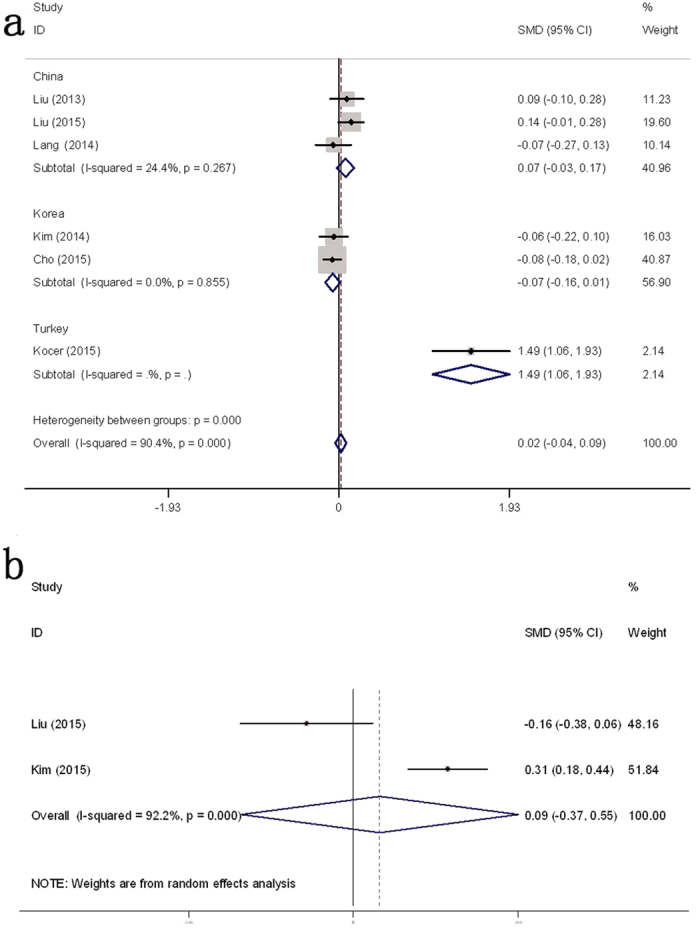
Subgroup analysis of forest plots of preoperative NLR in DTC patients compared with those with benign nodules. (**a**) NLR difference between DTC patients and those with benign nodules stratified by country. (**b**) NLR difference in PTC patients stratified by age, <45 y and ≥45 y.

Then we conducted subgroup analyses based on confounders, such as study location and tumor type. Stratified by location, we found that for patients in China^4^,^14^,^16^ the pooled MD was 0.07 (95% CI: −0.03 to 0.17, I^2^ = 24.4%, *P* = 0.267; Fig. 3a). For patients in Korea^12^,^17^, the pooled MD was −0.07 (95% CI: −0.16 to 0.01, I^2^ = 0.0%, *P* = 0.855). Subgroup analyses by papillary thyroid carcinoma (PTC)^12^,^13^,^14^,^16^,^17^ indicated that the pooled mean difference was 0.05 (95% CI: −0.05 to 0.08, I^2^ = 90.4%, *P* < 0.001; Fig. 2b).

### NLR in patients with PTC stratified by age

Two studies^14^,^15^ contained NLR data for PTC patients aged <45 years (496 cases) or ≥45 years (891 cases). The pooled MD between the age groups was 0.09 (95% CI: −0.37 to 0.55, I^2^ = 92.2%, *P* < 0.001; Fig. 3b).

### Heterogeneity

**Figure 4:**
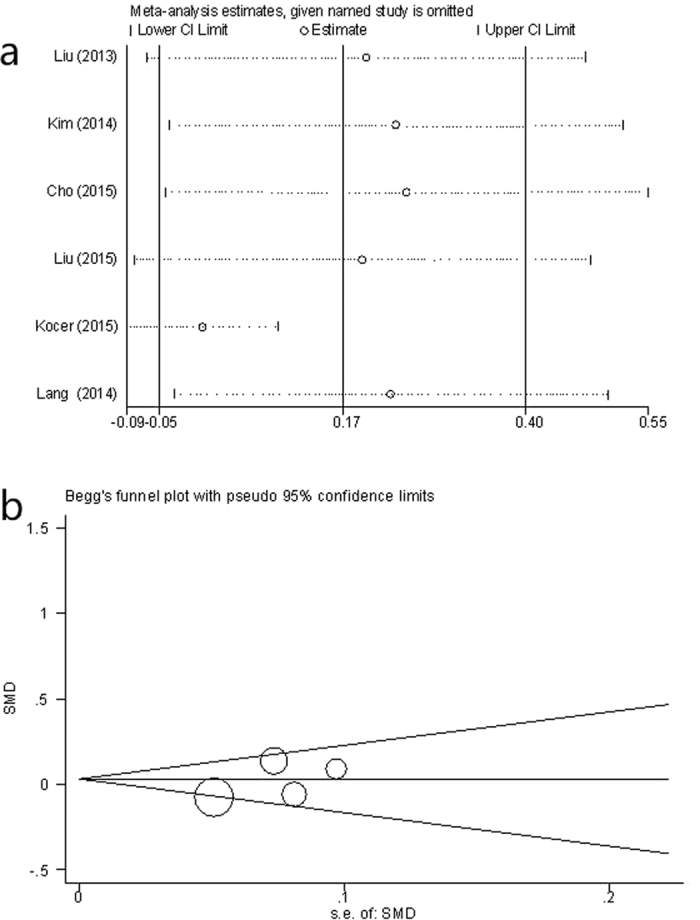
Sensitivity analysis and Funnel plots. (**a**) Sensitivity analysis on the association between NLR and thyroid nodule. (**b**) Funnel plots of studies included in the meta-analyses for thyroid nodules.

To investigate the influence of individual data sets on the pooled mean differences, each cohort included in our meta-analysis was deleted in turn in separate analyses. Results of sensitivity analyses indicated the robustness of our findings (Estimate: 0.17, 95% CI: −0.52 to 0.40) (Fig. 4a). By excluding one of the studies^13^, the heterogeneity was reduced significantly (I^2^ = 40%, *P* = 0.16) and the mean difference was nil (95% CI: −0.08 to 0.07).

### Publication bias

The publication bias estimate is mainly used to evaluate the reliability of the meta-analysis results, especially when a statistically significant difference is shown. The assessment of publication bias using Begg’s test suggested that was no significant publication bias in the studies (*P* = 0.26; Fig. 4b).

## Discussion

This meta-analysis aimed to examine the associations between pretreatment NLR and DTC. To the best of our knowledge, the present study is the first meta-analysis to investigate a potential association between them. Our study combined the outcomes of 6283 patients with thyroid nodules (4617 DTC and 1666 benign nodules) from 6 individual studies^4^,^12^,^13^,^14^,^16^,^17^. The meta-analysis showed that the pretreatment NLR values were not significantly different between patients with DTC and those with benign nodules. The subgroup and sensitivity analyses showed that the study of Kocer *et al*.^13^ was the source of heterogeneity, because its sample was much smaller than that of the others. The results were not weakened by subgroup analyses stratified by study location or tumor type. Although Kocer *et al*.’s^13^ report suggested that NLRs were significantly higher in patients with PTC than in patients with benign nodules, we noted that the age of the PTC patients (53.57 ± 13.32 y) appeared older than the age of patients with benign nodules (49.25 ± 13.15 y), although the article did not provide the *P*-value. These results suggest that an elevated NLR is neither a sensitive nor a specific indicator of progressing DTC in patients with goiters. Although Cho *et al*.^17^ pointed out that the NLR was a meaningful diagnostic tool for discriminating poorly differentiated thyroid cancer (n = 14) from anaplastic thyroid cancer (n = 7), their results were too limited by the small sample size and require further validation.

As reported in several studies, NLR correlated with thyroid cancer characteristics, such as tumor size, patient’s age, and the clinical stage parameters of thyroid cancer. Age is an important prognostic factor for patients with PTC^17^. Seretis *et al*.^11^, Liu *et al*.^14^, and Lang *et al*.^16^ found that older patients with PTC had significantly higher NLRs. However, Kim *et al*.^15^ found that the preoperative NLR was significantly lower in patients older than 45 years. Consequently, we pooled the only 2 studies that investigated age^14^,^15^ and found no difference in NLR value between patients aged younger or older than 45 years. Liu *et al*.^4^ found that the preoperative NLR correlated with DTC tumor size. Because there was limited data from the available studies, except regarding age, we did not conduct a pooled analysis of the correlation between elevated NLR and clinicopathological characteristics. However, Liu *et al*.^14^ and Kim *et al*.^15^ showed that tumor size, extra thyroidal invasion, and lymph node metastasis was not associated with the NLR value.

The prognosis of patients with cancer is not determined solely by tumor characteristics, but also by other factors related to the patient’s condition^12^. More recently, several meta-analyses reported the prognostic value of NLR in a variety of cancers^19^. Since PTCs have a favorable prognosis, 5 years may be considered too short to evaluate the prognostic implications^12^. Thus, there are too few studies to provide sufficient evidence for the prognostic value of NLR for PTC. Liu *et al*.^4^ pointed out that a high preoperative NLR was associated with a high American Thyroid Association (ATA) risk of recurrence in patients with differentiated thyroid cancer. With only 15 disease-specific events (1 distant metastasis and 14 locoregional recurrences), Kim *et al*.^12^ reported that in stage III-IV patients the 5-year disease-free survival rate was significantly worse in patients with NLR ≥ 1.5 than in those with NLR < 1.5. However, according to Lang *et al*.’s report^16^ a higher NLR did not predict a worse disease-free survival or higher risk of occult central nodal metastasis in cN0 PTC. This is not contrary to the fact that a higher NLR may imply a poorer tumor profile and prognosis. One possible reason may be because the prognosis of DTC, especially for PTC, is generally very good and so its NLR is expected to be low and within a narrow range.

In addition to the intrinsic defects associated with meta-analyses^19^, the present study is limited by significant heterogeneity among the studies that was due to sample size. Secondly, we did not analyze a correlation between the NLR and clinicopathological parameters of patients, such as lymph node metastasis, grade of differentiation, or tumor stage. Although age may affect the NLR, only 2 studies reported the relevant information, which was pooled with significant heterogeneity. Finally, in several reports the prognostic value of NLR for a variety of cancers was reported, and the data specifically for DTC was insufficient to analyze the prognostic value of NLR.

Despite the several limitations, our meta-analysis also had some advantages. First, the results were similar when the data were analyzed by subgroup, which indicates robustness of the statistical power of the results. Secondly, the sensitivity analysis did not significantly alter the results, supporting their stability. In addition, all the Newcastle-Ottawa quality assessment scale (NOS) scores of study quality were ≥7, which also supports the creditability of the results of the meta-analysis. Finally, although there were only 7 studies, the pooled sample was very large (nearly 7000 cases).

In conclusion, our results indicate that the NLR of patients with DTC is not significantly different from that of patients with benign nodules. An elevated NLR seems not a reliable indicator of progressing DTC in patients with goiters. In addition, there was no difference in NLR value between patients aged less than 45 years and those older than 45 years. More well-designed and large-scale investigations are warranted to understand better the value of the NLR in the prognosis of DTC.

## Methods

### Publication search

This meta-analysis was executed in accordance with guidelines of the Preferred Reporting Items for Systematic Reviews and Meta-Analyses (PRISMA)^20^. The following databases were searched for eligible studies up to 6 April 2016, without language restrictions: PubMed, Cochrane Library, EMBASE, Wan Fang Database, VIP Database for Chinese Technical Periodicals, and China National Knowledge Infrastructure. The terms used in the systematic literature search were: (“thyroid cancer” or “thyroid carcinoma”) and (“NLR” or “neutrophil to lymphocyte ratio” or “neutrophil lymphocyte ratio” or “‘neutrophil-lymphocyte-ratio”). Other resources were manually searched for any relevant records that were potentially missed. Moreover, reference lists of retrieved articles were reviewed for any studies that were not identified from the preliminary literature searches.

### Study selection criteria

Studies were included if they met the following criteria: patients with thyroid cancer that was histopathologically confirmed; NLR values were reported or obtained by communication with the authors; and evaluated the correlation between NLR and thyroid cancer. Articles were excluded from the meta-analysis if they were: letters; conference abstracts; editorials; review articles; not in the English or Chinese languages; animal, or irrelevant; or contained overlapping or duplicate data.

### Quality assessment

The quality of the studies was assessed according to NOS^18^. This scale includes 3 aspects of evaluation: selection, comparability, and outcomes between the case and control groups. Studies that scored ≥6 were considered high quality. Any disagreement between the investigators was resolved by discussion.

### Data extraction

Two investigators independently evaluated and extracted the data. All studies were double-checked by both investigators, and disagreements were resolved by consensus. The extracted data elements of this review were: publication details (first author’s last name, publication year, and origin of the studied population); characteristics of the studied population (sample size, age, and stage of disease); and mean and standard deviation of the NLR for thyroid nodules. If data for the mean and standard deviation were not available, we contacted the author for the original data.

### Statistical analysis

We used the mean differences with 95% CIs to calculate continuous data and identify differences in NLR values among groups of patients. A test of heterogeneity of the pooled results was performed using I^2^. I^2^ > 50% was considered significant heterogeneity. Both random effects (DerSimonian-Laird method) and fixed-effects (Mantel–Haenszel method) models were used to generate the pooled mean differences and 95% CIs. Because of a tendency toward heterogeneity among primary studies, the random-effects model was chosen because it is usually more conservative. We also investigated reasons for inter-study heterogeneity using subgroup analyses.

Sensitivity analyses were conducted to evaluate the stability of the results. Publication bias was evaluated using Begg’s funnel plot. All statistical tests were 2-sided and the significance level was set at 5% (*P* < 0.05). All analyses were conducted using STATA 12.0 software (STATA, College Station, TX).

## Additional Information

**How to cite this article**: Liu, J.-F. *et al*. Association between neutrophil-to-lymphocyte ratio and differentiated thyroid cancer: a meta-analysis. *Sci. Rep.* **6**, 38551; doi: 10.1038/srep38551 (2016).

**Publisher's note:** Springer Nature remains neutral with regard to jurisdictional claims in published maps and institutional affiliations.

## Figures and Tables

**Table 1:** Characteristics of included studies*.

First author (y)	Ref.	Country	Duration	Subjects, n	Thyroid nodule type	DTC	NOS	Pre-operative sample collection
Cho (2015)	18	Korea	2004–2009	3870	NH, TFA, PTC, MTC, TFC, HCC, PDTC, ATC	I-IV	8	7–14 d
Kim (2014)	12	Korea	2005–2012	6212	WN, BN, PTC	I-IV	8	1 m
Kim (2015)	14	Korea	2011–2013	1066	PTC	I-IV	8	1 d
Kocer (2015)	13	Turkey	2012–2014	232	MNG, LT, LT-PTC, PTC	NR	7	NR
Lang (2014)	17	China	2004–2012	469	BN, PTC, PTC	I-IV	7	1 d
Liu (2013)	4	China	2008–2010	577	BN, DTC	I-IV	8	1 d
Liu (2015)	15	China	2008–2013	841	PTC, NG, TA	I-IV	8	1–3 d

ATC, anaplastic thyroid cancer; BN, benign nodule; DTC, differentiated thyroid carcinoma; HCC, Hurthle cell carcinoma; LT, lymphocytic thyroiditis; MNG, multinodular goiters; MTC, medullary thyroid cancer; NH, nodular hyperplasia; NR: not reported; PDTC, poorly differentiated thyroid cancer; PTC, papillary thyroid cancer; TFA, thyroid follicular adenoma; TFC, thyroid follicular cancer; WN, without nodules.

^*^All studies are retrospective.
